# Vermicompost Alters Soil Microbial Communities and Decomposition but Increases Nitrate Leaching in Tropical Sugarcane

**DOI:** 10.1002/ece3.72925

**Published:** 2026-01-23

**Authors:** A. D. Canning

**Affiliations:** ^1^ Centre for Tropical Water and Aquatic Ecosystem Research (TropWATER), James Cook University Townsville Australia

**Keywords:** compost, diffuse pollution, microbial processes, nutrient cycling, organic soil amendments, soil metabarcoding

## Abstract

Agricultural intensification has elevated nitrogen losses that threaten water quality and farm efficiency. Organic amendments such as vermicompost are promoted as tools to enhance soil health and reduce fertiliser demand, yet their effects under tropical field conditions remain uncertain. A six‐week field trial in Australian sugarcane soil tested factorial combinations of vermicompost and nitrogen fertiliser. Metabarcoding, trait‐based analysis and structural equation modelling were used to assess soil biodiversity, decomposition and nitrate leaching. Bacterial genera diversity increased in the control soils during early wet‐season recovery but rose less in vermicompost‐amended soils. Vermicompost also shifted bacterial community composition and accelerated decomposition, while fungal and nematode responses were small over the short timeframe. Despite these microbial shifts, vermicompost increased nitrate leaching through a strong direct effect, with indirect pathways via fungi and decomposition remaining weak and uncertain. These findings show that vermicompost can stimulate microbial activity yet still exacerbate nitrogen losses in clay soils with moderate nutrient retention during high rainfall. Management strategies that align organic amendments with crop uptake or combine them with stabilising materials may help capture microbial benefits while reducing off‐site impacts.

## Graphical abstract

A six‐week field trial in Australian sugarcane soil used metabarcoding, trait‐based analysis and structural equation modelling to test how vermicompost affects soil biodiversity, decomposition and nitrate leaching under tropical conditions. Vermicompost shifted bacterial community composition and accelerated decomposition, yet increased nitrate leaching through strong direct effects rather than microbial pathways. These findings show that vermicompost can stimulate microbial activity while still exacerbating nitrogen losses in clay soils during high rainfall, suggesting management strategies should align organic amendments with crop uptake timing.

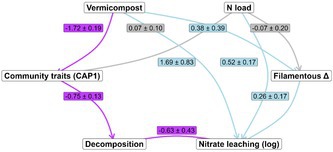

## 1 Introduction

The intensification of agriculture has substantially increased fertiliser use worldwide, resulting in nitrogen losses that degrade water quality and contribute to greenhouse gas emissions. Excess nitrogen from fertilisers can leach into waterways, driving eutrophication in freshwater and coastal ecosystems, or volatilise as nitrous oxide, a potent greenhouse gas (Bowles et al. 2018; Schulte‐Uebbing et al. 2022; Shibata et al. 2017). Schulte‐Uebbing et al. (2022) estimate that a safe planetary boundary for the annual nitrogen surplus is 64% lower than the global nitrogen surplus realised in 2010. Nitrogen loss extent depends on fertiliser management practices, soil properties, and the capacity of soil biota to regulate nitrogen cycling processes such as immobilisation, mineralisation, nitrification and denitrification (López‐Aizpún et al. 2021; Zhang and Yu 2020). In addition to environmental impacts, high nitrogen loss can reduce farm efficiency and profitability (Gu et al. 2023; Pannell 2017). Gu et al. (2023) found that implementing a suite of nitrogen management practices across global croplands could reduce nitrogen losses by 30%–70% and yield a global societal benefit of $476 ± 123 billion (USD 2015) while costing $19 ± 5 billion (USD 2015). Identifying strategies to improve nutrient retention while maintaining crop productivity is therefore critical for improving both ecological health and agricultural profitability (Liu et al. 2024; Losacco et al. 2021; Mahmud et al. 2021).

Regenerative farming approaches that aim to rebuild soil organic matter, increase biological activity and enhance nutrient retention are increasingly proposed to reduce fertiliser demand and losses (Lal 2020; Rehberger et al. 2023; Rhodes 2017). The use of organic amendments, such as composts, manures, slurries and biochar, is common in regenerative farming, and it can increase soil organic carbon, microbial biomass, cation exchange capacity and water retention while providing a slow‐release nutrient supply (Crystal‐Ornelas et al. 2021; Tully and McAskill 2020). However, outcomes are context dependent, with variability in crop responses and nutrient dynamics depending on amendment type, soil texture, climate and management (Crystal‐Ornelas et al. 2021; Urra et al. 2019). Risks associated with organic amendments include the potential mobilisation of excess nitrogen, heavy metals, or contaminants such as antibiotics and microplastics, which may undermine environmental and human health (Urra et al. 2019).

Vermicompost, produced through the bio‐oxidation of organic matter by earthworms and associated microorganisms, is increasingly recognised as a promising organic amendment for improving soil health and nutrient management. It is nutrient rich, finely structured, and biologically active, and has been shown to enhance soil quality, improve nutrient availability, stimulate plant growth, increase resistance to pests and diseases, and reduce reliance on synthetic fertilisers (Lim et al. 2015; Oyege and Balaji Bhaskar 2023; Rehman et al. 2023; Yatoo et al. 2021). A meta‐analysis by Blouin et al. (2019) indicated that vermicompost application enhanced plant performance, with average increases of 26% in commercial yield, 13% in total biomass, 78% in shoot biomass, and 57% in root biomass. These effects are linked to the provision of readily available organic matter, microbial inocula, and bioactive compounds such as hormones, humic substances and enzymes that stimulate microbial activity and plant–soil interactions (Rehman et al. 2023). Vermicompost also improves soil physical and chemical properties by enhancing aggregation, porosity and water retention, and by increasing soil organic matter and cation exchange capacity (Lim et al. 2015; Vuković et al. 2021). The combination of both physical and nutritional changes can reshape soil microbial assemblages and the prevalence and activity of functional guilds within them, such as nitrifiers, denitrifiers, mycorrhizal fungi and antagonistic microbes, and consequently (Bouchtaoui et al. 2024; Tully and McAskill 2020). In turn, these shifts may alter nutrient cycling alter higher trophic levels such as nematodes (Amaya‐Gómez et al. 2025; Broz et al. 2016; Jouquet et al. 2011). If vermicompost shifts the nitrogen balance toward lower fixation and more immobilisation then nitrogen losses could reduce, consequently retaining more nitrogen in the soil for the benefit of plants and the broader environment. Alternatively, if vermicompost stimulated mineralisation or increases solute mobility then nitrogen losses may increase (Bagheri et al. 2021; Broz et al. 2016; Jouquet et al. 2011). It is, therefore, critical that the use of vermicompost is well understood in terms of how soil type, fertiliser regime, application rate and climate shape the outcomes to ensure its use matches with desired outcomes (Oyege and Balaji Bhaskar 2023; Vuković et al. 2021).

In Australia's Great Barrier Reef (GBR) catchment, large reductions of nitrogen runoff are required to improve the health and resilience of the GBR (Brodie et al. 2017). Much of the nitrogen lost within the catchment comes from upstream nitrogen‐intensive sugarcane farming (Davis et al. 2017). Despite substantial investment in fertiliser efficiency programmes, nitrogen losses remain high, highlighting the need for novel management practices that both sustain production and reduce nutrient leakage (McCloskey et al. 2021; van Grieken et al. 2019). Organic amendments such as vermicompost have the potential to enhance soil health and microbial function, benefiting farmers and aquatic ecosystems across the globe (Shu et al. 2022; Wu et al. 2023). Yet their role in mediating nitrogen losses in fields with synthetic fertiliser application is seldom experimentally tested in the field, despite indications that organic soil amendments may improve nitrogen use efficiency and reduce losses (Phillips et al. 2022; Tang et al. 2022; Toda et al. 2023).

To better understand the soil microbe and nitrogen dynamics of vermiculture use in tropical agriculture with concurrent nitrogen fertiliser use, this study conducted a field trial in sugarcane in the Australian tropics. Specifically, this study aimed to: (i) evaluate the effects of vermicompost treatment and nitrogen loading on soil community diversity, measured as changes in bacterial, fungal and nematode alpha diversity; (ii) assess how vermicompost and nitrogen loading influence shifts in microbial and nematode community composition (beta diversity), and to identify associated functional traits and environmental drivers; and (iii) test hypothesised causal pathways linking vermicompost treatment, microbial traits, litter decomposition, soil nutrient dynamics and nitrogen leaching using structural equation modelling.

## 2 Methods

### 2.1 Experiment

Nitrate leaching from topsoil was examined over 6 weeks following nitrate application at five loading rates, across randomised soil plots (4 × 4 m) established within a 2‐ha coastal sugarcane field near Forrest Beach, Queensland. The dominant geology at the site is Quaternary coastal and estuarine sediments, with soils largely composing clay on river alluvial plains (Grundy et al. 2015; McKenzie et al. 2012). The experiment was conducted between 11 November and 23 December 2022 to coincide with the onset of the monsoonal rains, when nutrient mobilisation typically begins, and concluded prior to the onset of flooding. Half of the plots were randomly assigned to receive vermicompost at an application rate of 10 t per ha while the other half served as controls.

Vermicompost was applied as a commercial worm casting product (Verasoil Organic Worm Castings, Verasoil Australia). According to manufacturer‐supplied quality data (Verasoil 2021), the material was a mature, high‐humic compost (humic acids 8.9%; fulvic acids 2.4%; C:N 8.8; pH 7.4; EC 2.8 dS/m; total organic carbon 9.5%) with microbial biomass of 51.6 mg/kg, a fungi: bacteria ratio of 2.4 and strong microbial maturity and disease‐suppression indicators.

Nitrogen was applied as potassium nitrate (KNO_3_) dissolved in 10 L of water and evenly distributed across the trial plots using an electronic spray gun. Application rates were 0, 25, 50, 75 and 100 kg N/ha, with plots replicated at three random locations within each of the vermicompost‐amended and control treatments, giving a total of 30 plots. The 10 t ha^−1^ vermicompost rate reflects mid‐range values reported in sugarcane and field cropping trials (Djajadi et al. 2020; Roberts et al. 2007; Singh et al. 2007; Wako and Muleta 2022), while the nitrogen range (0–100 kg N ha^−1^) spans locally relevant split‐application fertiliser inputs near the onset of the wet season to reduce losses and better match crop uptake (Calcino et al. 2022; Skocaj et al. 2013; Thorburn et al. 2011). Soil chemistry and soil biota were assessed in each plot immediately prior to nitrate application and again 6 weeks later, while nitrate leaching was assessed passively over the 6 weeks using the ion‐exchange method described below.

### 2.2 Soil Characterisation and Decomposition

To characterise baseline soil conditions, composite 0–30 cm samples were collected from each plot at the beginning and end of the experiment (Table 1). Within each plot, five soil cores were taken at random, pooled, homogenised and submitted to the Environmental Analysis Laboratory (Southern Cross University) for a standard agronomic panel. Analyses included organic carbon, C:N ratio, ammonium, nitrate, Colwell P, potassium, sulphur, exchangeable cations (Ca, Mg, Na), aluminium, CEC, pH (water and CaCl_2_), electrical conductivity and chloride. A single deep core from the centre of the experimental area provided a field description of the soil profile to 1.6 m. The upper 30 cm comprised a clay‐loam surface (0–10 cm) grading to silty light clay by 20–30 cm, underlain by light–medium clay. Moist Munsell colours (10YR 3/2 to 10YR 5/3) indicated moderately well‐drained clayey soils.

**TABLE 1:** Baseline soil parameters (0–30 cm) across all plots prior to experimental applications.

Parameter	Mean	SD	Min	Median	Max
Aluminium (KCl)	2.01	0.92	0.2	2.3	3.4
Ammonium (mg/kg)	3.40	1.08	1.9	3.2	6.4
C:N Ratio	14.13	1.25	12	14	18
CEC cmol(+)/kg	9.13	1.77	6.87	8.715	13.6
Ca:Mg Ratio	0.90	0.37	0.45	0.825	1.7
Chloride (mg/kg)	153.43	159.84	31	110	860
EC (dS/m)	0.28	0.17	0.09	0.24	0.72
Magnesium	2.87	0.58	2.1	2.75	4
Nitrate (mg/kg)	37.78	34.52	1.2	29.5	100
Organic Carbon (%)	2.64	0.52	1.94	2.56	4.34
Phosphorus (Colwell)	68.33	43.78	20	58	180
Potassium	0.62	0.39	0.2	0.505	1.6
Sodium	0.89	0.41	0.46	0.77	2.2
Sulphur (KCl‐40)	39.73	26.42	14	28.5	120
pH (CaCl_2_)	4.18	0.26	3.9	4.1	4.9
pH (Water)	4.90	0.17	4.3	4.9	5.3

As nitrogen enrichment can accelerate decomposition processes, wood decomposition was assessed concurrently by burying wooden stirring sticks (~15 × 1.5 × 0.2 cm) under 10 cm of soil at three random positions per plot. Mass loss was calculated by comparing initial oven‐dried mass (3 days at 70°C) to final rinsed and oven‐dried mass at the end of the experiment.

Nitrate leaching from the topsoil (30 cm depth) during the study was estimated using ion‐exchange resins. Prior to nitrogen application, a single resin bag was buried at the centre of each plot. Ion‐exchange resins are now widely applied in both agricultural (Hess et al. 2020; Karhu et al. 2021; Woodward et al. 2022) and ecological research (Borken and Matzner 2004; Guerrieri et al. 2021; Ibrahim et al. 2021) as a tool for monitoring nitrate loss and have proven effective relative to traditional lysimeter approaches (Pampolino et al. 2000; Wey et al. 2021). Unlike lysimeters, which capture leachate at discrete points in time, resins accumulate nitrate passively and continuously, providing an integrated measure between sampling events. This is especially advantageous in environments where nitrate loss is highly variable. At the flood‐prone study site, resins also offered a practical alternative to in situ sensors by lowering the risk of damage or loss and reducing the need for regular site access (Qian and Schoenau 2002).

Resin bags (50 cm^2^ mesh pouches) each contained 50 g of nitrate‐selective ion‐exchange resin (Resinex NR‐1) and were inserted 30 cm below intact soil via a replaced soil core following G. Singh et al. (2018). Resinex NR‐1, a premium‐grade cross‐linked polystyrene divinylbenzene resin, was chosen because of its strong nitrate selectivity, even in sulphate‐rich soils, and its low susceptibility to nitrate displacement by organic matter (Edgar and Boyer 2022). In the laboratory, nitrate was extracted from the resins with 200 mL of 2 M KCl under agitation (2 h at 300 rpm). Extracts were then analysed for nitrate using UV spectrophotometry (American Public Health Association, American Water Works Association and Federation 2017) and then corrected using laboratory calibration curves to account for incomplete recovery.

### 2.3 Soil Metabarcoding

Soil samples were sent to Metagen Australia for metabarcoding of bacterial/archaeal (16S), fungal (18S) and nematode (18S) communities using the primer sets Pro341F/Pro805R (Takahashi et al. 2014), NF1/18S2rB (Porazinska et al. 2009) and Nemf/18Sr2b (Sikder et al. 2020), respectively. DNA was extracted from 10 g subsamples following a modified modular universal protocol (Sellers et al. 2018), with humic acids removed and DNA purified using SPRI beads (Oberacker et al. 2019). Dual‐indexed amplicons were generated using a two‐step PCR protocol adapted from the Illumina 16S Metagenomic Sequencing Library Preparation workflow, pooled in equimolar concentrations, and sequenced on an Illumina MiSeq platform (2 × 300 bp) at the University of Queensland. Raw reads were demultiplexed with DeML (Renaud et al. 2015), and sequence variants were inferred with DADA2 (Callahan et al. 2016) in R. Low‐quality reads and chimaeras were filtered using default error thresholds and the ‘consensus’ method, respectively. Taxonomic assignment was performed using the naïve Bayesian classifier, with SILVA v138.2 (Quast et al. 2013) for 16S rRNA and PR2 v4.12 (Guillou et al. 2013) for 18S rRNA.

### 2.4 Alpha Diversity

Amplicon sequence variant (ASV) tables for bacteria (16S rRNA), fungi (18S rRNA) and nematodes (18S rRNA) were processed separately. Using the microeco package in R 4.4.3 (Liu et al. 2021; R Core Team 2024), datasets were filtered to the target kingdom/phylum and collapsed to the genus level before normalisation with cumulative sum scaling (CSS). Shannon diversity indices were calculated for each sample, and diversity change was expressed as the difference between post‐ and pre‐ samples (Post—Pre) for each plot. Linear models were then fitted to test for effects of vermicompost treatment and nitrogen loading on changes in Shannon diversity.

### 2.5 Beta Diversity

Beta diversity was analysed separately for bacteria, fungi, and nematodes using the microeco package in R 4.4.3 (Liu et al. 2021; R Core Team 2024). Community data were normalised with cumulative sum scaling (CSS) and ordinated using principal coordinates analysis (PCoA) based on Bray–Curtis dissimilarities across pre‐ and post‐treatment samples. Within‐plot changes in community composition were quantified as the difference in PCoA scores between post‐ and pre‐application samples. Treatment effects of vermicompost and nitrogen loading on multivariate community shifts were tested with PERMANOVA and distance‐based redundancy analysis (db‐RDA) implemented in vegan (Dixon and Palmer 2003; Oksanen et al. 2007). Associations between db‐RDA axes and soil properties or functional traits were evaluated using the envfit function with 999 permutations and the Benjamini‐Hochberg procedure applied. Soil variables included nitrate leaching rate, decomposition rate and changes in total carbon, total nitrogen, and the C:N ratio relative to pre‐experiment values.

Functional traits were assessed through the relative abundance of taxa assigned to ecological guilds relevant to nitrogen cycling: bacterial traits from FAPROTAX (Louca et al. 2016; Sansupa et al. 2021), fungal guilds from FUNGuild (Nguyen et al. 2016), and nematode traits from Nemaplex (H Ferris 1999). Nematodes were further categorised by feeding group (bacterivores, fungivores, plant parasites, omnivores, predators) and by coloniser–persister (CP) scale scores from 1 (r‐strategists) to 5 (K‐strategists), which reflect life‐history strategies and disturbance tolerance (Bongers and Bongers 1998; Ferris et al. 2001). Low‐CP bacterivores typically dominate nutrient‐rich soils and enhance nitrogen mineralisation via microbial grazing, whereas higher‐CP nematodes are indicative of stable, resource‐limited conditions with slower nutrient turnover (Bongers and Bongers 1998; Ferris and Bongers 2006; Ferris et al. 2001).

Differential responses of microbial genera were analysed using the trans_diff function in microeco with the glmm_beta method, which fits beta‐distributed GLMMs via glmmTMB. This approach appropriately models proportional amplicon data and accommodates repeated measures, random effects and treatment × time interactions, making it more suitable for our experimental design than count‐based methods such as DESeq2 or ALDEx2. Models tested vermicompost and nitrogen load effects across pre‐ and post‐treatment sampling with waypoint included as a random effect (Brooks et al. 2017; McGillycuddy et al. 2025).

### 2.6 Structural Equation Model

Hypothesised causal pathways linking vermicompost addition, microbial functional traits, and nitrogen loss were evaluated using structural equation modelling (SEM). Observed variables in the SEM were vermicompost treatment, nitrogen loading, bacterial community composition (CAP1), filamentous fungal growth, litter mass loss and log‐transformed nitrate leaching. Vermicompost and nitrogen loading were allowed to shift microbial traits (CAP1 and filamentous fungi). In turn, shifts in bacterial community structure were hypothesised to influence decomposition (mass loss), while changes in filamentous fungi were expected to influence nitrate leaching. Direct pathways from vermicompost and nitrogen loading to nitrate leaching were also included to capture treatment effects not mediated by microbes (e.g., altered nutrient availability). The direct vermicompost → leaching path was specified separately from indirect microbial pathways, allowing total, direct and indirect effects to be partitioned.

Collinearity was assessed using variance inflation factors (VIFs) from linear models fitted to each endogenous regression. Microbial predictors and nitrogen loading showed low VIF values (< 2), while vermicompost and mass loss showed higher VIF values (~7). These arise from expected structural correlation in the causal chain (vermicompost → CAP1 → mass loss → leaching) rather than problematic multicollinearity.

A Bayesian SEM was fitted using four chains (burn‐in = 1000, 4000 posterior samples) with the blavaan package in R 4.4.3 (Merkle and Rosseel 2018; R Core Team 2024) and then plotted using DiagrammeR (Iannone and Roy 2016). Model adequacy was assessed using posterior predictive checks. The posterior predictive *p*‐value was 0.00, which is typical for small‐sample SEMs with non‐normal variables. Local model fit was evaluated using posterior predictive residuals based on standardised covariance discrepancies; all residuals were small (EAP range −0.84 to −0.10) with no concentrated pattern of misfit, indicating that the model structure was adequate for summarising the main pathways. For each path, posterior means and the posterior probability of being positive or negative were extracted to describe effect magnitude and direction.

## 3 Results

### 3.1 Nitrate Leaching

Post‐treatment nitrate leaching averaged 15.7 ± 73.9 kg N ha^−1^ in control plots. Vermicompost addition was associated with an increase of 186.9 ± 73.9 kg N ha^−1^ (*p* = 0.018), while nitrogen load was not associated with leaching (*β* = 0.82 ± 1.05, *p* = 0.439). The model explained 20.6% of the variation in nitrate leaching (*F*(2, 27) = 3.50, *p* = 0.044).

### 3.2 Bacteria

**FIGURE 1:**
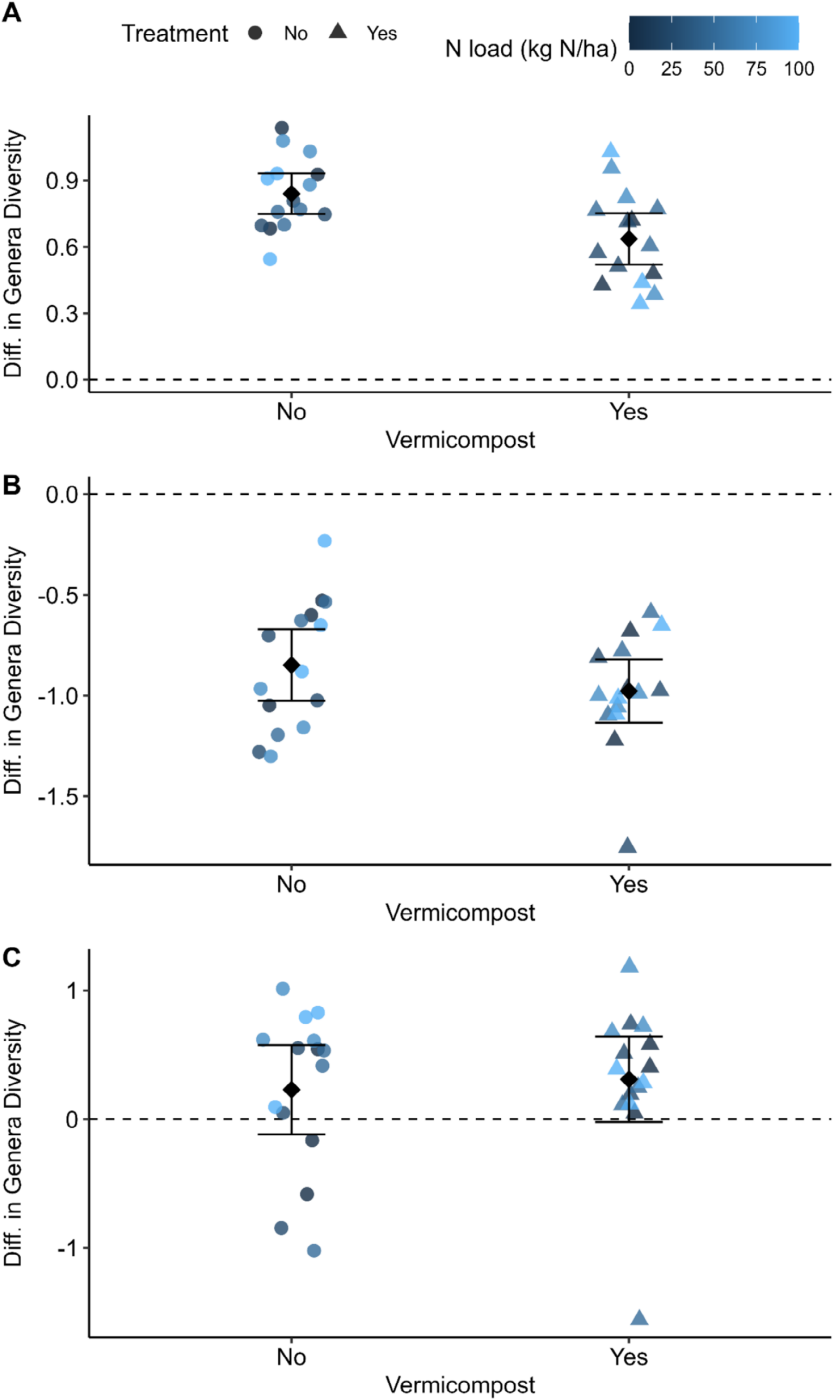
The difference (post experiment–pre experiment) in Shannon's genera diversity of (A) bacteria, (B) fungi and (C) nematodes for each plot, classified by whether vermicompost was applied, and coloured by the nitrogen load (kg N/ha) applied. Error bars indicate one standard deviation either side of the mean.

**FIGURE 2:**
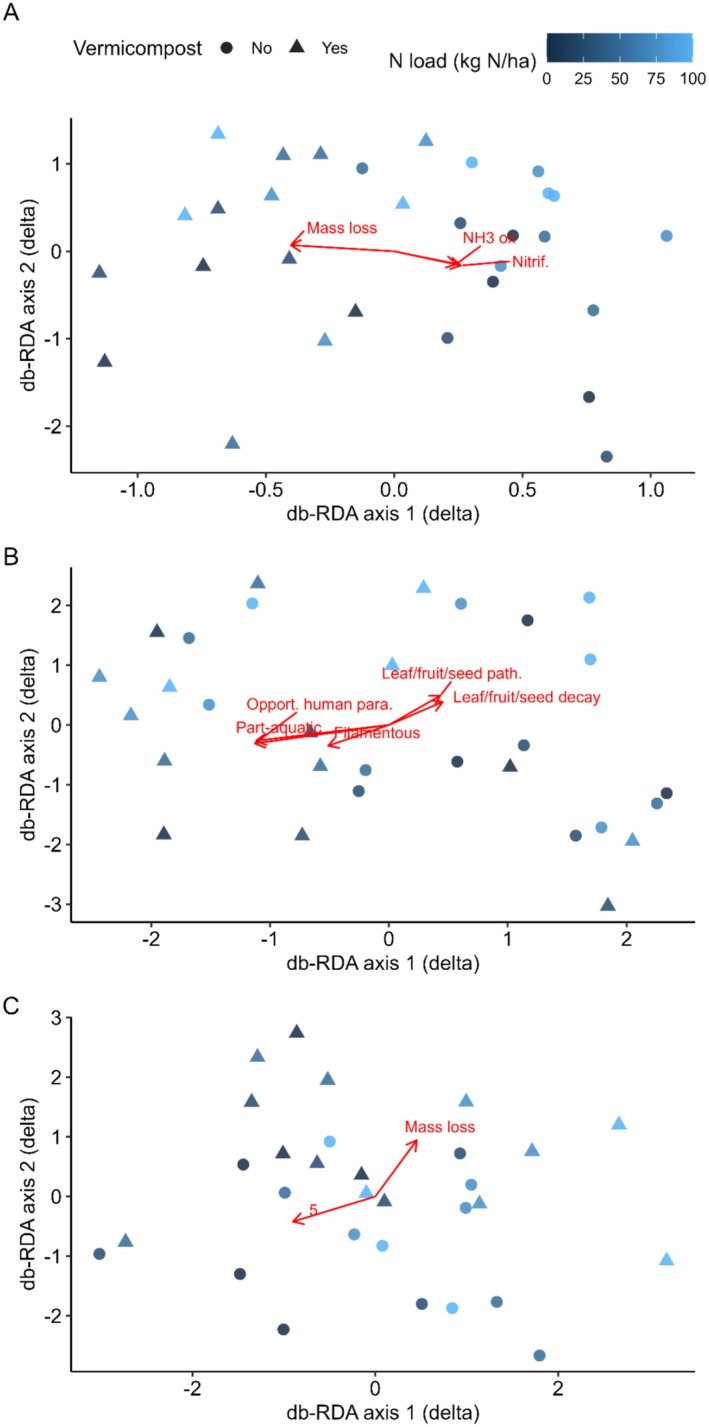
Distance‐based redundancy analysis (db‐RDA) plots of the difference (post experiment—pre experiment) in PCoA scores using Bray–Curtis Dissimilarity and CSS transformation of (A) bacteria, (B) fungi and (C) nematodes for each plot. Shapes indicate whether vermicompost was applied, and colours indicate the nitrogen load (kg N/ha) applied. Fitted overlays indicate variables where correlation *p*‐values were < 0.05 following Benjamini‐Hochberg correction.

**FIGURE 3:**
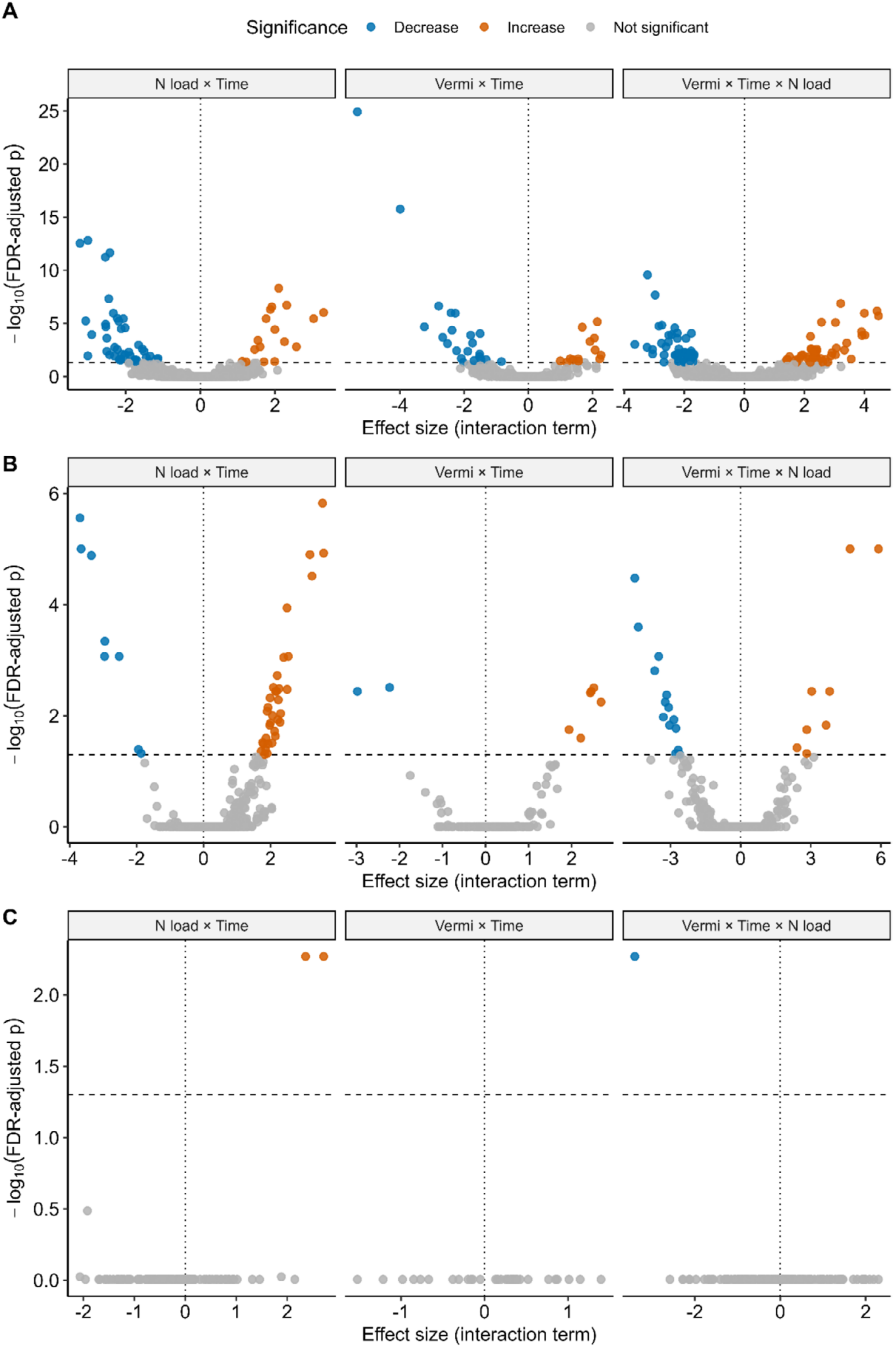
Volcano plots showing results from genus‐level glmmTMB models for (A) bacteria, (B) fungi and (C) nematodes. Each point represents a genus, with model effect sizes (x‐axis) plotted against significance (−log_10_ FDR‐adjusted *p*; y‐axis). Horizontal dashed lines mark the 0.05 threshold.

The change (Post—Pre) in Shannon's diversity of bacteria genera was lower in the plots with vermicompost than those without (*β* = −0.21 ± 0.07, *p* = 0.006), but was unaffected by nitrogen load (*β* = 0.0005 ± 0.0010, *p* = 0.653), with the overall model explaining 24.9% of the variance (*F*_2,27_ = 4.48, *p* = 0.021; Figure 1). A PERMANOVA indicated that the change in community composition, measured as the difference (Post—Pre) in PCoA scores with Bray–Curtis dissimilarity (Figure 2), was associated with vermicompost (*R* ^2^ = 0.10, *F* = 3.23, *p* = 0.001) but not nitrogen load (*R* ^2^ = 0.03, *F* = 0.87, *p* = 0.69). Compositional shifts were correlated with mass loss (*r* ^2^ = 0.64, *p* = 0.001), and the change (post—pre) in relative abundance of aerobic ammonia oxidising bacteria (*r* ^2^ = 0.44, *p* = 0.002) and nitrifying bacteria (*r* ^2^ = 0.46, *p* = 0.002). Positive and negative responses were detected across bacterial genera under all interaction terms, with 55 showing large positive shifts and 34 showing large negative shifts under vermicompost × time (Figure 3; Table 2; File S1). The strongest increases were observed in *Gammaproteobacteria*, *Longispora* and *Azospirillum*, whereas *Blastocatella* and *Pirellula* showed the largest declines. Amongst genera associated with nitrification, *Azospirillum* showed a strong positive response to vermicompost × time (File S1), whereas effects on *Nitrospira* and *Pseudomonas* were weaker and not statistically supported.

**TABLE 2:** Summary of genus‐level differential abundance responses to vermicompost × time effects (beta‐GLMM).

Group	Genera tested	Number with large positive effect	Number with large negative effect	Genera with most positive effect	Genera with most negative effect
Bacteria	696	55	34	*Gammaproteobacteria, Longispora, Azospirillum, Micromonosporaceae, Pantoea*	*Blastocatella, Pirellula, Novosphingobium, Virgisporangium, Duganella*
Fungi	204	7	17	*Tremellomycetes, Teratosphaeriaceae, Fabrella, Mycosphaerella, Cryptomycotina_XX*	*Exobasidiomycetes_X, Tilletiopsis, Xylaria, Coniosporium, Sordaria*
Nematodes	36	0	0		

*Note:* Genera were classified as showing a large increase or decrease where effect estimates exceeded |0.5| and were statistically significant (FDR < 0.05). Only taxa with resolved genus names are included. Full model outputs are provided in File S1.

### 3.3 Fungi

**FIGURE 4:**
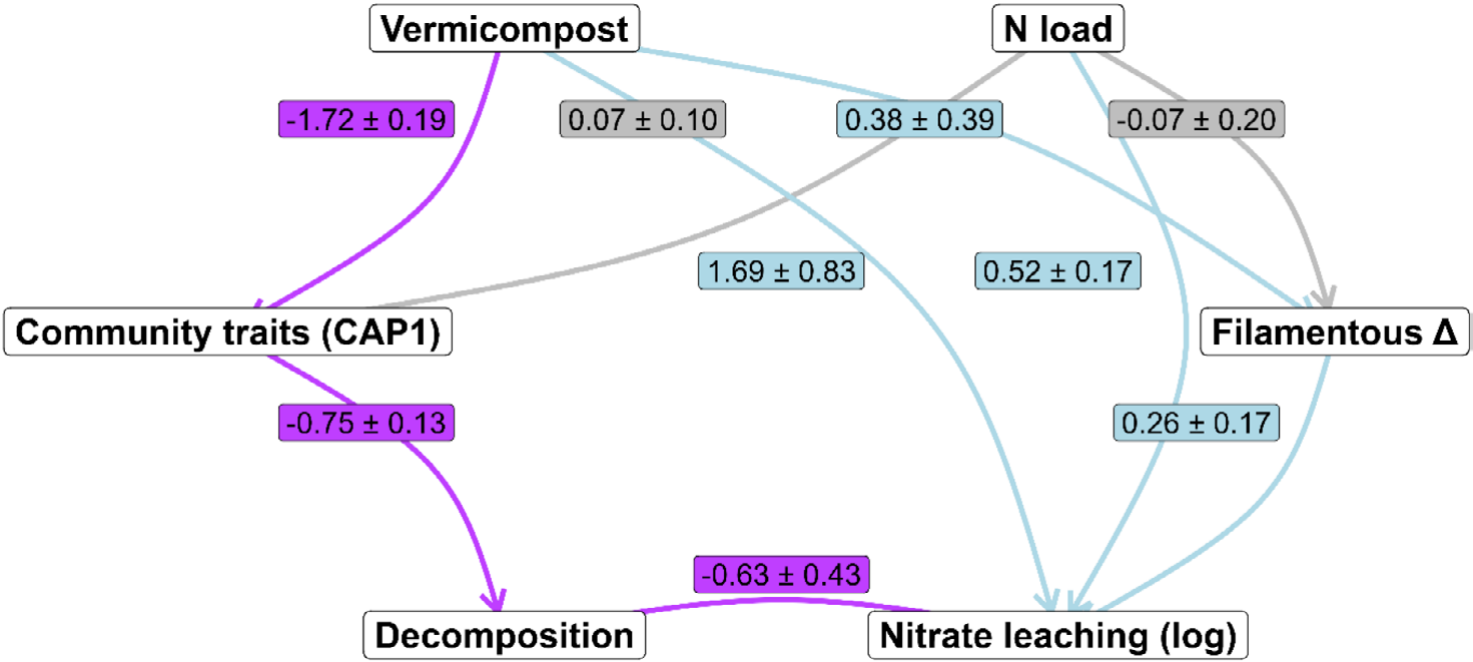
Bayesian structural equation model (SEM) showing hypothesised pathways linking vermicompost addition and nitrogen load to microbial traits, decomposition and nitrate leaching. Nodes represent observed variables: Vermicompost, N load (kg N ha^−1^), bacteria community ordination CAP1 axis site scores, change in filamentous fungi (Filamentous Δ), decomposition (mass loss), and nitrate leaching (kg N ha^−1^). Arrows indicate direction of influence, with colours denoting the posterior probability of effect direction: Light blue for positive relationships (≥ 80% probability), purple for negative relationships (≥ 80% probability), and grey for uncertain associations. Edge labels show posterior mean ± standard error of regression coefficients.

The change (Post—Pre) in Shannon's diversity of fungi genera did not differ by either vermicompost use (*β* = −0.13 ± 0.111, *p* = 0.259) or nitrogen load (*β* = 0.0007 ± 0.001, *p* = 0.639), with the overall model being insignificant (*F*_2_,_27_ = 0.78, *p* = 0.47; Figure 1). A PERMANOVA indicated that the change in community composition, measured as the difference (Post—Pre) in PCoA scores with Bray–Curtis dissimilarity, was not associated with either nitrogen load (*R* ^2^ = 0.06, *F* = 1.78, *p* = 0.094) or vermicompost (*R* ^2^ = 0.02, *F* = 0.53, *p* = 0.89; Figure 2). Compositional shifts were significantly associated with partly aquatic taxa (*r* ^2^ = 0.44, *p* = 0.007), opportunistic human parasites (*r* ^2^ = 0.44, *p* = 0.007), filamentous mycelial growth forms (*r* ^2^ = 0.29, *p* = 0.032), leaf/fruit/seed pathogens (*r* ^2^ = 0.30, *p* = 0.032), and leaf/fruit/seed decay taxa (*r* ^2^ = 0.28, *p* = 0.042). No environmental variables remained associated after BH correction. Fungal responses were more limited, with 7 genera showing large increases and 17 showing large decreases under vermicompost × time (Figures 3 and 4; Table 2; File S1). Increases were most pronounced in *Tremellomycetes* and *Fabrella*, while declines were strongest in taxa such as *Tilletiopsis* and *Xylaria*.

### 3.4 Nematodes

The change (Post—Pre) in Shannon's diversity of bacteria genera was not associated with either vermicompost (*β* = −0.08 ± 0.22, *p* = 0.714) or nitrogen load applications (*β* = 0.004 ± 0.003, *p* = 0.145), with the overall model being insignificant (*F*_2_,_27_ = 1.20, *p* = 0.32; Figure 1). A PERMANOVA indicated that the change in community composition, measured as the difference (Post—Pre) in PCoA scores with Bray–Curtis dissimilarity, was not associated with either vermicompost (*R* ^2^ = 0.02, *F* = 0.5, *p* = 0.98) or nitrogen load (*R* ^2^ = 0.04, *F* = 1.07, *p* = 0.317; Figure 2). Compositional shifts were correlated with mass loss (*r* ^2^ = 0.41, *p* = 0.005) and the change (post—pre) in relative abundance of feeding group five (animal predators; *r* ^2^ = 0.40, *p* = 0.007). Nematodes showed no genera with large positive or negative responses under vermicompost × time (Figure 3; Table 2; File S1), indicating negligible short‐term shifts in nematode composition.

### 3.5 Structural Equation Model

Vermicompost reduced bacterial community composition scores (CAP1; est. = −1.72, 95% CI: −2.10 to −1.35), while nitrogen loading had little influence on CAP1 (est. = 0.07, 95% CI: −0.12 to 0.26). Effects on filamentous fungal growth were uncertain for both vermicompost (est. = 0.38, 95% CI: −0.40 to 1.13) and nitrogen loading (est. = −0.07, 95% CI: −0.46 to 0.32). CAP1 was negatively associated with decomposition (mass loss; est. = −0.76, 95% CI: −1.01 to −0.50). Log‐transformed nitrate leaching increased with filamentous fungal abundance (est. = 0.26, 95% CI: −0.07 to 0.59) and showed a weak negative association with mass loss (est. = −0.63, 95% CI: −1.48 to 0.22). Vermicompost had a positive direct effect on nitrate leaching (est. = 1.69, 95% CI: 0.05 to 3.33), and nitrogen loading also increased leaching (est. = 0.52, 95% CI: 0.19 to 0.86). Indirect effects of vermicompost were small and uncertain, both through filamentous fungi (est. = 0.10, 95% CI: −0.16 to 0.36) and through decomposition (est. = −0.82, 95% CI: −1.97 to 0.34). The total effect of vermicompost on nitrate leaching remained positive (est. = 0.97, 95% CI: 0.09 to 1.85). All parameters showed R‐hat = 1.00, indicating good chain convergence.

## 4 Discussion

### 4.1 Soil Biotic Responses

Vermicomposts are widely marketed as enhancers of microbial diversity, although evidence for these claims is mixed and the idea of what constitutes a ‘beneficial’ microbe is subjective. In this experiment, bacterial genera diversity increased over time in the unamended control soils. This rise reflects the expected wet‐season recovery phase in these clay soils, driven by rainfall, fresh residue inputs and post‐fertiliser disturbance, which collectively stimulate a short‐term expansion in microbial richness. These background dynamics formed the benchmark for interpreting treatment responses. Soils receiving vermicompost showed a smaller increase in Shannon diversity than the controls, while nitrogen additions alone had no detectable influence. These background dynamics provided the comparative benchmark against which treatment effects were evaluated. Soils receiving vermicompost showed a smaller increase in Shannon diversity than the controls, while nitrogen additions alone had no detectable influence. Vermicompost also shifted bacterial community composition, with changes linked to overall mass loss and to the relative abundance of aerobic ammonia oxidisers and nitrifiers, although these latter groups were more strongly associated with plots that did not receive vermicompost. By contrast, fungal and nematode genera diversity showed no significant treatment responses, and overall community composition did not differ by vermicompost or nitrogen addition. Nonetheless, the *envfit* correlations revealed patterns within these assemblages. For fungi, partly aquatic taxa, opportunistic human parasites, and filamentous growth forms tended to align with plots receiving vermicompost, while leaf‐ and fruit‐associated decay and pathogenic fungi aligned more with untreated soils. However, the effect sizes were small, so these associations should be interpreted as minor shifts rather than strong treatment‐driven patterns. For nematodes, compositional variation was associated with mass loss, which pointed more toward vermicompost treatments, and with the relative ASV abundance of predators (feeding group 5) being more aligned with low‐nitrogen plots. These functional associations suggest that vermicompost primarily influenced processes linked to organic matter turnover rather than broad gains in diversity, while nitrogen inputs subtly shaped higher trophic interactions without restructuring communities.

The limited and sometimes counterintuitive effects observed here are consistent with wider evidence that claims about vermicompost as a microbial inoculant require careful scrutiny. Many studies emphasise the diversity of microbes within vermicompost itself and assume these organisms establish in soils once applied (Domínguez et al. 2019; Pereira et al. 2023; Vuković et al. 2021). However, microbial assemblages may be altered during storage and handling, and once added they encounter established soil communities and environmental filters that limit successful colonisation. Moreover, the habitat provided by mineral soils under field conditions differs considerably from the homogeneous and nutrient‐rich substrate of vermicompost and is subject to fluctuating moisture, temperature, tillage, root exudates and resource availability (Philippot et al. 2021, 2024; Tecon and Or 2017). For these reasons, microbial diversity measured in vermicompost cannot be assumed to directly translate into increased diversity in soils.

Where field and greenhouse trials have been conducted, the outcomes remain variable. Vermicompost has been linked to altered fungal composition and improved crop yields in greenhouse cucumber and long‐term tomato monocultures (Zhao et al. 2020; Zhao et al. 2017), yet other studies report only very small or statistically unsupported changes in diversity. For example, slight increases in rhizobacterial diversity in Gujarat soils were correlated with Mn content, but the differences were numerically small and lacked error estimates, with more robust findings instead emerging from the functional traits of individual isolates rather than community‐level shifts (Pandya et al. 2014).

By contrast, established research consistently shows that field microbial communities are structured primarily by soil properties. For example, soil pH is a dominant predictor across ecosystems (Philippot et al. 2024), physical structure regulates predator–prey dynamics and mobility (Erktan et al. 2020), and soil texture influences fungal richness, with coarse textures favouring greater richness and finer textures promoting filamentous taxa and enhanced carbon degradation potential (Xia et al. 2020). At finer scales, spatial heterogeneity in pores, diffusion gradients, and water films creates diverse microsites that sustain coexistence (Tecon and Or 2017). These well‐documented drivers are unlikely to have shifted substantially with vermicompost addition, which helps explain why vermicompost produced only modest and assemblage‐specific effects in this experiment. The soils were clay textured, with moderate CEC, and likely provided a stable physical and chemical matrix that limited strong treatment‐driven shifts in microbial diversity. However, longer term applications of vermicompost have observed improved aggregate stability driven by an increase in humic substances and polysaccharides (Cruz et al. 2024), which could then have more substantial influence on shaping the microbial communities. This highlights the need for caution when interpreting broad claims that vermicompost universally enhances soil microbial diversity; outcomes will be context dependent and likely require long‐term use to alter communities via soil habitat. When fertiliser is applied as split applications, it is typically reapplied at roughly 6‐week intervals, and nutrient mobilisation peaks at the onset of the wet season, so the experiment was timed to capture that early window when mobilisation is greatest. The results therefore reflect short‐term microbial responses; slower processes such as fungal colonisation, nematode turnover, and seasonal nutrient cycling develop over longer periods, and multi‐season trials would be required to resolve extended community trajectories.

### 4.2 Direct Nitrate Leaching From Vermicompost Outweighs Microbial Mediation

Vermicompost addition substantially increased nitrate leaching, whereas nitrogen loading alone had little effect. This outcome is consistent with column studies showing greater nitrate and dissolved solids loss from vermicompost‐amended soils, particularly under saturated flow (Bagheri et al. 2021). Vermicompost typically contains more soluble N and other labile nutrients than conventional composts, reflecting accelerated mineralisation and humification by earthworms and microbes (Lim et al. 2015). Studies have also shown that mixing vermicompost with inorganic fertiliser can boost microbial biomass, soil C and available N, lowering the C:N ratio and increasing the pool of N at risk of leaching (Li et al. 2019). At the same time, vermicompost can enhance soil structure, porosity, aggregate stability, and cation exchange capacity, properties that may under some conditions reduce nutrient losses (Cruz et al. 2024). The outcome is therefore context dependent: vermicompost may promote immobilisation and long‐term retention of fertiliser N in soil organic matter, or accelerate nitrate leaching when its high N content and rapid nitrification exceed soil retention capacity (Amaya‐Gómez et al. 2025; Broz et al. 2016; Sande et al. 2024). Although several nitrifier‐associated genera increased under vermicompost (Table 2; File S1), their contribution to nitrate loss was minimal over the short timeframe of this experiment. In particular, *Azospirillum* showed a strong positive response to vermicompost over time, whereas effects on other nitrifier‐linked genera such as *Nitrospira* and *Pseudomonas* were weaker and not statistically supported. The SEM indicated that indirect effects via filamentous fungi and decomposition were small and uncertain, while the direct vermicompost pathway to nitrate leaching was consistently strong. Microbial processes may become more influential over longer timescales once communities stabilise and immobilisation capacity increases, but this was not yet apparent here.

In this experiment, nitrate leaching was greater (and more variable) under vermicompost additions, most likely because of high rainfall combined with acidic (pH 4–5), clay soils with a moderately low cation exchange capacity (9.1 cmol(+)/kg) and a moderate C:N ratio (~14). Although clays typically retain nutrients better than sands, these soils offered limited microbial immobilisation potential, and their fine structure may have promoted preferential flow and rapid nitrate transport under rainfall. The structural equation model supported this interpretation, showing that vermicompost had a strong direct positive effect on nitrate leaching while indirect pathways through microbes and decomposition were comparatively small and uncertain. Vermicompost slightly increased filamentous fungi growth (which was positively associated with leaching), shifted bacterial community composition (CAP1), and increased nitrifier abundance, and promoted decomposition (which were negatively associated with leaching). Overall, the dominant mechanism was a direct effect of vermicompost inputs on nitrate mobilisation and loss, with microbial sinks insufficient to offset this pathway. Given that the trial was conducted in a single sugarcane field, extrapolation to other soils, crops, or climates should be done cautiously.

In regions like northern Australia, where sugarcane and other tropical crops intersect with sensitive waterways and reef systems, the potential for organic amendments to exacerbate nitrate losses has direct relevance for water quality targets and sustainable intensification strategies (Davis et al. 2017). From a management perspective, vermicompost may be best applied sparingly, outside of periods of heavy rainfall, and in synchrony with crop uptake. Future trials should run over longer periods and test approaches such as blending vermicompost with stabilising amendments (e.g., biochar), while also assessing how different feedstocks shape microbial responses and nutrient losses.

### 4.3 Conclusion

This study highlights the dual role of vermicompost as both a soil biostimulant and a potential driver of nutrient losses. In this tropical sugarcane system, vermicompost clearly altered bacterial community structure and decomposition processes, yet it also increased nitrate leaching, with direct effects outweighing microbial buffering. These results caution against broad claims of vermicompost as universally beneficial and emphasise the importance of context, including soil properties, rainfall patterns, and nutrient cycling dynamics. While vermicompost can enhance microbial activity and nutrient cycling, in instances where rainfall is high and microbial immobilisation capacity is low it may instead accelerate nutrient losses with implications for downstream water quality. Future research should use long‐term experiments to explore management options such as synchronising applications with crop uptake, blending vermicompost with stabilising materials like biochar, and diversifying feedstocks to improve retention. Vermicompost's contrasting effects on microbes and nutrient losses reinforce the need to manage organic amendments in ways that strengthen agroecosystem resilience and safeguard downstream environments.

## Author Contributions

**A. D. Canning:** conceptualization (lead), data curation (lead), formal analysis (lead), investigation (lead), methodology (lead), project administration (lead), software (lead), visualization (lead), writing – original draft (lead), writing – review and editing (lead).

## Funding

The author has nothing to report.

## Conflicts of Interest

The author declares no conflicts of interest.

## Supporting information


**Data S1:** ece372925‐sup‐0001‐FileS1.xlsx.

## Data Availability

Raw data is available from https://doi.org/10.6084/m9.figshare.30758732.
